# Detecting Effect of Levodopa in Parkinson’s Disease Patients Using Sustained Phonemes

**DOI:** 10.1109/JTEHM.2021.3066800

**Published:** 2021-03-17

**Authors:** Nemuel D. Pah, Mohammod A. Motin, Peter Kempster, Dinesh K. Kumar

**Affiliations:** 1 Electrical Engineering DepartmentUniversitas Surabaya 128202 Surabaya60293Indonesia; 2 School of EngineeringRMIT University 5376 MelbourneVIC3000Australia; 3 Department of Electrical and Electronic EngineeringRajshahi University of Engineering and Technology 166216 Rajshahi6204Bangladesh; 4 Monash Health 2538 ClaytonVIC3168Australia

**Keywords:** Dysarthria, drug response, Parkinson’s disease, sustained phonemes, voice analysis

## Abstract

Background: Parkinson’s disease (PD) is a multi-symptom neurodegenerative disease generally managed with medications, of which levodopa is the most effective. Determining the dosage of levodopa requires regular meetings where motor function can be observed. Speech impairment is an early symptom in PD and has been proposed for early detection and monitoring of the disease. However, findings from previous research on the effect of levodopa on speech have not shown a consistent picture. Method: This study has investigated the effect of medication on PD patients for three sustained phonemes; /a/, /o/, and /m/, which were recorded from 24 PD patients during medication *off* and *on* stages, and from 22 healthy participants. The differences were statistically investigated, and the features were classified using Support Vector Machine (SVM). Results: The results show that medication has a significant effect on the change of time and amplitude perturbation (jitter and shimmer) and harmonics of /m/, which was the most sensitive individual phoneme to the levodopa response. /m/ and /o/ performed at a comparable level in discriminating PD-*off* from control recordings. However, SVM classifications based on the combined use of the three phonemes /a/, /o/, and /m/ showed the best classifications, both for medication effect and for separating PD from control voice. The SVM classification for PD-*off* versus PD-*on* achieved an AUC of 0.81. Conclusion: Studies of phonation by computerized voice analysis in PD should employ recordings of multiple phonemes. Our findings are potentially relevant in research to identify early parkinsonian dysarthria, and to tele-monitoring of the levodopa response in patients with established PD.

## I. Introduction

Parkinson’s disease (PD) is the second most common neurodegenerative disorder [1]. With aging populations, its prevalence is expected to increase. The motor deficits of PD are caused by degeneration of the dopamine-producing (dopaminergic) neurons in the substantia nigra region of the brain. Pathological changes are present in other neuronal populations as well, explaining the development of various non-motor impairments in PD [2]. Most diagnoses are based on clinical detection of motor signs—the presence of two or more of tremor, rigidity, bradykinesia, or postural impairment [3]. Confirmatory evidence can be provided by dopamine transporter scanning, though this test is not widely available across the world. Other medical imaging modalities lack sensitivity. There is a need for biomarkers that can, with high reliability, recognize PD before overt motor signs appear.

The Movement Disorder Society Unified Parkinson’s Disease Rating Scale Part III (MDS-UPDRS-III) [4] is the standard tool for objective measurement of parkinsonian motor disability. However, scoring requires clinical observations and has the potential limitations of subjectivity, clinician bias, and inter-rater variability [5]. Consequently, there is some loss of sensitivity for early stage diagnostics, for monitoring disease progression, and for assessing the effectiveness of medication or other therapies [6]. The requirement for regular clinical visits can be burdensome in some circumstances, and there is a need for an objective measure of PD symptoms that is suitable for telehealth applications. The use of gait analysis [7] and handwriting have been proposed [8], but these require specialized equipment.

One of the early symptoms of PD is change in voice, which can precede other motor features [9]. Voice testing has been proposed for early diagnosis of the disease, or to monitor its progression [10]. Human speech is an overtrained and habitual response that requires fine-motor control, cognitive abilities, auditory feedback, and muscle strength [11]. Parkinsonian dysarthria can be characterized by reduced vocal tract loudness, reduced speech prosody, imprecise articulation, significantly narrower pitch range, longer pauses, vocal tremor, breathy vocal quality, harsh voice quality, and disfluency [12].

Studies on the voice or speech parameters can be divided into four groups based on the analyzed aspect: phonatory, articulatory, prosodic, and linguistic [13]. The study of articulatory, prosodic, and linguistic aspects [14] involves more complex and broad factors such as the psychology, linguistics, and cognitive conditions of patients, and this makes it difficult to diagnosis. On the other hand, phonatory aspects of voice are less obscured by these conditions. Phonation relates to the glottal source and resonant structures of the vocal tract and has greater potential for reliable diagnose of PD.

Numbers of studies have investigated the voice parameters obtained from sustained phonemes to determine the differences between PD patients and healthy participants [15]–[19]. Behroozi and Sami [20] introduced a multi-classifier framework to separate PD patients from healthy controls. The use of deep learning has also been applied for the classification of voice recordings [21]. Vaiciuknas *et al.* [12] investigated the strategy for PD screening from sustained phoneme parameters and text-dependent speech modalities. Voice analysis has also been proposed for estimating the severity of the disease. Tsanas *et al.* [22] investigated the relationship between speech signal parameters and motor disability score of PD patients. Perez *et al.* [23] developed an automatic feature extraction to diagnose PD and to track its progression. Khan *et al.* [24] evaluated PD severity based on vocal function assessment from audio recordings.

The voice features that have shown a significant difference between the voice of healthy and PD patients are pitch frequency, jitter, shimmer, and harmonics to noise ratio [9]. However, these parameters can also be affected by other factors such as age, gender, and ethnicity, and this can result in poor reliability. The pitch frequency, $f_{0}$, is the fundamental frequency of the vocal cords when producing a sound or phoneme and varies with sex, age, and health conditions. Jitter is the perturbation of the glottal vibration period which is affected by the diminished motor control, rigidity, and tremor of the larynx. Shimmer, the amplitude perturbation, is related to the glottal resistance and increases due to lack of control of the voice box and the breathing muscles. Harmonics to noise ratios (HNR and NHR) are the ratios between the periodic (voiced) and non-periodic (noise) component of the speech. These indicate the relative harmonic strength which is reduced with diminished glottal vibration. Low HNR is an indicator of the existence of dysarthria. Studies have reported the use of other features such as the fractal dimension (FD) [25] linear predictive model (LPM) [13], multivariate deep features [21], and entropy [25]. Many of these studies have shown these features are very effective in differentiating between the voices of PD and healthy people.

The motor symptoms of PD are managed by dopaminergic pharmacological treatments, of which levodopa is the most effective and widely used. Most patients improve on levodopa, though one weakness of the drug is the tendency for an unstable, fluctuating response to develop after a number of years [26]. Careful balancing of levodopa dosage and addition of other agents are often required to counteract these motor fluctuations. This is often a trial-and-error process, which may require a patient to undertake multiple visits to their neurologist [27]. Computerized analysis of speech could be useful for remotely monitoring the medication effects in PD patients. However, the results of studies that have evaluated the effect of medication on speech and voice parameters in PD are inconsistent, even contradictory [28]. The study by Rusz *et al.* [9] showed that levodopa might improve the consonant articulation in the early stages of PD. Elfmarkova *et al.* [29] found that levodopa only partially improves speech prosody in some patients. Contradicting these are the findings of Tykalova *et al.* [30] who reported an increase in dysfluent speech after 3 – 6 years of dopaminergic treatment compared to a drug-naive condition. Cusnie-Sparrow [31] found that the magnitude of the levodopa response may increase with increasing severity of the voice quality symptoms. Skodda *et al.* [32] studied the short and long-term dopaminergic effects on dysarthria in early Parkinson’s disease. They found that none of the parameters of phonation, intonation, articulation, and speech velocity improved significantly in the “on” state. Ho *et al.* [33] reported that there was no reliable or meaningful improvement in speech with the use of levodopa. However, the study did not use any of the above phonatory parameters. Instead, they used average intensity, intensity decay, and duration of speech within one breath envelope.

Studies that have primarily focused on phonation in PD do not show a consensus about levodopa effect. Sanabria *et al.* [34] found some decrease in jitter, fundamental frequency and harmonic-to-noise ratio in response to levodopa medication. However, they found no significant differences in shimmer. Goberman *et al.* [35] did not find any significant differences between PD and healthy groups in the fundamental frequency variability of prolonged vowels. They also found that the group differences between PD patients before and after medication was small. The study of Fabbri *et al.* [36] found no significant effect of levodopa on speech or voice. De Letter *et al.* [37] studied the changes of phonatory speech characteristics across a levodopa dose cycle. They investigated several respiratory, articulatory, prosodic measures, as well as phonatory features such as pitch, jitter, shimmer, harmonics to noise ratio. They found that the majority of speech acoustic parameters do not vary significantly with levodopa. Notably, only a single sustained phonation, either /a/ or /i/ was used in the studies of Rusz *et al.* [9], Santos *et al.* [38], and De Letter *et al.* [37].

The aim of this study was to investigate the use of phonatory parameters to classify PD patients before and after levodopa medication. We examined the change in phonatory parameters by using a statistical hypothesis test and the Support Vector Machine (SVM) algorithm to separate the two PD medication states and the control groups. Three different sustained phonemes were considered: /a/, /o/ and /m/. These phonemes were selected to examine a range of voice production [11]. The vowel /a/, as in “car”, is an open-back or low vowel, which is produced while the jaw is widely open, with the tongue is inactive and positioned low in the mouth. The vibration of the vocal folds dominates the sound of the vowel. The vowel /o/, as in “oh”, is a closed-mid-back vowel, in which the back of the tongue is positioned mid-high towards the palate, and the lips are at a rounded position. The phoneme /m/ is a voiced nasal phoneme which is produced by the vibration of the vocal folds with the air flowing through the nasal cavity. Although all three phonemes require control of the respiratory and laryngeal vocal fold muscles, there are considerable differences in patterns of activation of the rostral muscles of articulation (of pharynx, tongue, jaw and lips). Observations on a selection of voice parameters will reveal the effect of PD and its medication on each of these phonemes. Besides the statistical analysis, the machine learning approach was used to investigate the possible non-linear separation between the two classes.

## II. Methods

### A. Participants

Twenty-four PD patients (13 males and 11 females) were recruited from the Movement Disorders Clinic at Monash Medical Centre. All had been diagnosed within the last ten years and complied with the Queen Square Brain Bank criteria for idiopathic PD [39]. The presence of any advanced PD clinical symptoms—visual hallucinations, frequent falling, cognitive disability, or need for institutional care—was an exclusion criterion [40]. Twenty-two healthy participants (12 males and 10 females) were recruited from several retirement centers.

PD participants were first assessed in a practically defined *off* state (PD-*off*) (fasting, with anti-parkinsonian medication withheld for at least 12 hours). They were retested in the *on* state (PD-*on*), taken to be the maximum improvement 30 – 90 minutes after a subject’s usual morning levodopa dose. Motor function in *off* and *on* states was scored by a neurologist on the MDS-UPDRS-III [4]. Cognitive function was scored on the Montreal Cognitive Assessment scale [41]. The mean levodopa equivalent daily dose, calculated using standard conversion factors, was 480 ± 296 mg/day [42]. Table 1 presents participants’ demographic and clinical information.

**TABLE 1:** Participants’ Demographics

	Control Subjects	PD Subjects	p-value
Number of subjects	22	24	
Age	66.30 ± 6.20	71.92 ± 7.07	0.008
PD-off MDS-UPDRS-III score	N/A	25.54 ± 8.78	1.42e-05 (PD-off vs PD-on)
PD-on MDS-UPDRS-III score	N/A	19.33 ± 9.30	
MoCA	28.30 ± 1.34	27.25 ± 2.67	0.118
Duration of disease (years)	N/A	5.29 ± 2.99	

The study protocol was approved by the ethics committee of Monash Health, Melbourne, Australia (LNR/16/MonH/319) and RMIT University Human Research Ethics Committee, Melbourne, Australia (BSEHAPP22-15KUMAR). Before the experiments, written informed consent was obtained from all the participants.

### B. Voice Recording

Three sustained phonemes /a/, /o/, and /m/ were recorded from each participant. The participants were instructed to pronounce the vowel for as long as it was comfortable, with their natural pitch and loudness.

The phonemes were recorded using Samson-SE50, an omnidirectional head-worn microphone. The recordings were saved into a single-channel uncompressed WAV format with a sampling rate of 48 kHz and a 16-bit resolution. Each recording contained one single sustained phoneme of 5.1 to 38.6 seconds duration as shown in Table 2. There were 60 seconds of relaxation time between each recording. The recording was performed in a noise-restricted room. The de-identified data is available on RMIT website and has been reported earlier [25].

**TABLE 2:** Duration of the Recordings

	min – max (mean ± std) in sec		
	Control	PD-off	PD-on
/a/	5.1–19.1 (11.3±2.7)	5.5–18.2 (9.7±3.2)	5.3–14.4 (9.8±2.5)
/o/	6.7–23.3 (12.2±3.6)	5.8–38.6 (12.9±6.7)	5.2–16.8 (11.3±3.1)
/m/	5.7–16.5 (11.7±2.9)	5.8–23.8 (11.3±4.0)	5.6–15.3 (10.2±2.4)

### C. Parameter Extraction

MATLAB2018b (MathWorks) was used for all analyses. Each recording was manually segmented to eliminate any unwanted sections such as silent pieces and the voice of the instructor. Based on the assumption that vowels correspond to largely stationary signals, and the need for more samples for the purpose of cross-validation, each recording was divided into ten segments of 0.5 seconds each, and the jitter, shimmer, pitch, and harmonics parameters of each segment was calculated. Each segment was considered as an individual example.

The features of each segment were calculated using Praat [43], a publicly available software for analyzing, synthesizing, and manipulating speech. The first step for feature extraction was to locate the time instances ($t_{i}$) of the pulses in the recording that represent the glottal vibration. The instantaneous period of the glottal wave ($T_{i}$) was calculated as the difference between subsequent instances of the pulses, $T_{i} =t_{i+1}-t_{i}$.

Four jitter parameters were extracted from the recordings: jitter absolute (*abs*), jitter relative (*rel*), relative average perturbation (*rap*), and period perturbation quotient-*5 (ppq5).* The *rap* and *ppq5* are the perturbation of the difference between $T_{i}$ and the moving average of $T_{i}$ with a window size of 3 and 5, respectively. The equation to calculate the four jitter parameters [44] are shown in equations 1 to 4:

\begin{align*} Jitter\left ({abs }\right)=&\frac {1}{N-1}\sum \nolimits _{i=1}^{N-1} \left |{ T_{i+1}-T_{i} }\right |\tag{1}\\ Jitter\left ({rel }\right)=&\frac {\frac {1}{N-1}\sum \nolimits _{i=1}^{N-1} \left |{ T_{i+1}-T_{i} }\right |}{\frac {1}{N}\sum \nolimits _{i=1}^{N} T_{i}}\tag{2}\\ Jitter(rap)=&\frac {\frac {1}{N-2}\sum \nolimits _{i=2}^{N-1} \left |{ T_{i}-\left ({\frac {1}{3}\sum \nolimits _{n=i-1}^{i+1} T_{n} }\right) }\right | }{\frac {1}{N}\sum \nolimits _{i=1}^{N} T_{i}}\tag{3}\\ Jitter(ppq5)=&\frac {\frac {1}{N-4}\sum \nolimits _{i=3}^{N-2} \left |{ T_{i}-\left ({\frac {1}{5}\sum \nolimits _{n=i-2}^{i+2} T_{n} }\right) }\right | }{\frac {1}{N}\sum \nolimits _{i=1}^{N} T_{i}}\tag{4}\end{align*}

Five shimmer parameters extracted from the segments are the absolute shimmer (in dB), the relative shimmer, *apq3, apq5*, and *apq11* measured in percentage. The *apq3, apq5*, and *apq11* are the perturbation of the difference between $A_{i}$ and the moving average of $A_{i}$ with a window size of 3, 5, and 11, respectively. The parameter calculations are described in equations 5 to 9.

\begin{align*} Shimmer\left ({abs,dB }\right)=&\frac {1}{N-1}\!\sum \nolimits _{i=1}^{N-1} \!\left |{ 20\ast \mathrm {log}\left ({\frac {A_{i+1}}{A_{i}} }\right) }\right | \\ \tag{5}\\ Shimmer\left ({rel }\right)=&\frac {\frac {1}{N-1}\sum \nolimits _{i=1}^{N-1} \left |{ A_{i+1}-A_{i} }\right |}{\frac {1}{N}\sum \nolimits _{i=1}^{N} A_{i}} \\ \tag{6}\\ Shimme(apq3)=&\frac {\frac {1}{N-2}\sum \nolimits _{i=2}^{N-1} \!\left |{ A_{i}-\!\left ({\frac {1}{3}\sum \nolimits _{n=i-1}^{i+1} A_{n} }\right) }\right | }{\frac {1}{N}\sum \nolimits _{i=1}^{N} A_{i}} \\ \tag{7}\\ Shimmer(apq5)=&\frac {\frac {1}{N-4}\sum \nolimits _{i=3}^{N-2}\! \left |{ A_{i}-\!\left ({\frac {1}{5}\sum \nolimits _{n=i-2}^{i+2} A_{n} }\right) }\right | }{\frac {1}{N}\sum \nolimits _{i=1}^{N} A_{i}} \\ \tag{8}\\ Shimmer(apq11)=&\frac {\frac {1}{N-10}\sum \nolimits _{i=6}^{N-5} \left |{ A_{i}-\left ({\frac {1}{11}\sum \nolimits _{n=i-5}^{i+5} A_{n} }\right) }\right | }{\frac {1}{N}\sum \nolimits _{i=1}^{N} A_{i}} \\{}\tag{9}\end{align*}

The pitch parameters are the mean, median, standard deviation, maximum, and minimum of the instantaneous pitch frequency $f_{0i} = 1/T_{i}$. The *HNR* and *NHR* were calculated based on the normalized autocorrelation function of the segment. $R_{xx}[T_{0}]$ is the peak next to the centre of $R_{xx}$ at a distance corresponding to the $T_{0}$ of the recording. The *HNR* and *NHR* were calculated as described in equations 10 and 11 [45], [46]:

\begin{align*} HNR=&10\ast log\frac {R_{xx}[T_{0}]}{1-R_{xx}[T_{0}]}\tag{10}\\ NHR=&1-R_{xx}[T_{0}]\tag{11}\end{align*}

**FIGURE 1.**
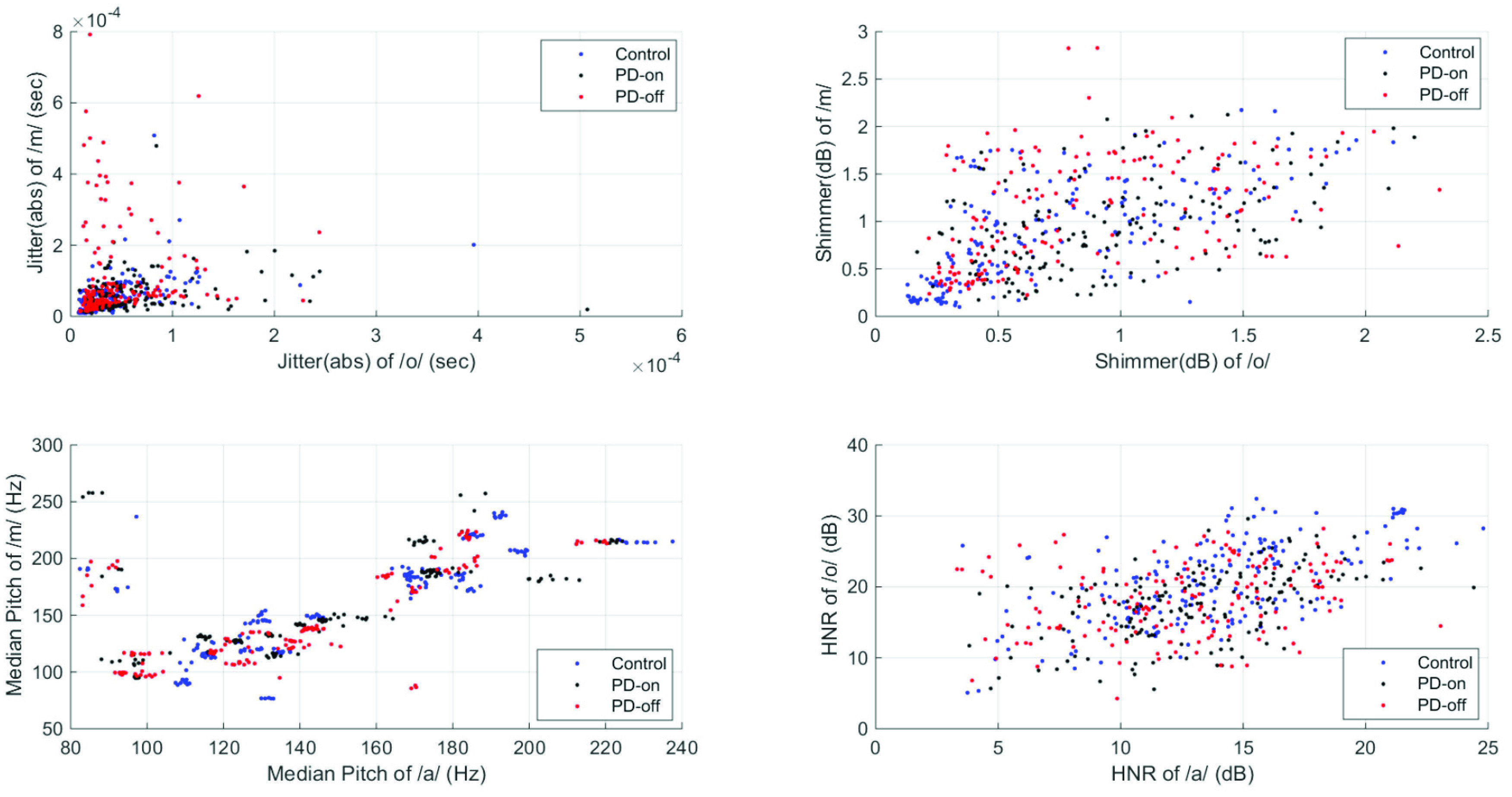
The scatter plot of some selected features. a) Jitter(abs), b) Shimmer(dB), c) Median Pitch, and d) HNR.

The scatter plots on Fig. 1 illustrate the distribution of the features for the different classes. The figure indicates that there is a high level of overlap between classes.

### D. Statistical Analysis

All the statistical analyses were performed using MATLAB2018b (MathWorks). The normality of the extracted parameters was examined with the Anderson-Darling test [47]. Statistical non-parametric Wilcoxon signed-rank test [48] was then applied to compare voice parameters between PD-*on* and PD-*off* to determine the effect of medication on the patient. Mann Whitney U-test [48] was used to compare the group differences for voice parameters between CO and PD-*off*, and CO and PD-*on.*

The $p$-values for age and MoCA scores were calculated using independent sample t-tests, while paired t-testing was used to compare PD-*off* and PD-*on* MDS-UPDRS-III scores.

The 95% confidence level was considered for the analysis and $p-value < 0.05$ indicated that the mean of the groups was significantly different.

### E. Machine Learning Based Classification

Support Vector Machines (SVM) [49] classifier with Gaussian kernel and fifth-order cross-validation model was used in this study. The Gaussian kernel was selected since it yielded the best result compared to the other kernels. It was trained to model the hyperplane that can separate the groups using the input as the extracted voice features. Seven SVMs were created to classify the groups. The input to the SVMs were the voice features described in Section II (c) with the exception of mean, median, max, and min of pitch features because of the known gender-based difference- the dataset was not suitable for testing based on the gender divide. The input to the first three SVMs was the parameters of phoneme /a/, /o/, and /m/, respectively. The input to the other three SVMs was the combination of two phonemes, /a/+/o/, /a/+/m/, and /o/+/m/. The parameters of all three phonemes were given as the input to the seventh SVM.

The size of dataset for CO subject was $220 \times 36$. The 220 corresponds to 22 subjects $\times \,\, 10$ segments/ subject. The 36 corresponds to $3 \times 12-12$ features for each phoneme /a/, /o/, /m/. The 12 features for each phoneme were: 4 jitters, 5 shimmers, std pitch, HNR, and NHR. Size of PDF and PDN dataset were $240 \times 36$, since we have 24 PD subjects.

For each SVM training, only 80% of the training sets (randomly picked) were used, while the other 20% were used for testing ($5^{\mathrm {th}}$ order validation). The above has now been inserted in the revised manuscript.

The classification was evaluated based on the true-positive (TP), true-negative (TN), false-positive (FP), and false-negative (FN). The Receiver Operating Characteristic (ROC) curve was generated, and the Area Under Curve (AUC) was calculated for each SVM model.

## III. Results

### A. Statistical Analysis

Anderson-Darling test confirmed that the voice parameters for the three groups and the three phonemes were not normally distributed and thus unsuitable for parametric test. Mann Whitney U test was used to test for group differences in each of the features. Wilcoxon signed-rank test was used to test the differences between dependent data of PD-*on* and PD-*off*.

#### 1) Wilcoxon Signed-Rank Test Between PD-on and PD-off

Table 3 presents the result of the Wilcoxon signed-rank test between PD-*on* and PD-*off*, and this identifies the features that are significantly changed by medication. It shows that *p-value* was less than 0.05 for most of the parameters of phoneme /m/. The jitter and harmonics parameters of phoneme /o/, as well as the pitch of phoneme /m/ were also changed due to medication.

**TABLE 3:** The Result of the Wilcoxon Signed-Rank Test Between PD-on and PD-off

Parameters	Phoneme /a/			Phoneme /o/			Phoneme /m/		
	Mean+Std		p-value	Mean+Std		p-value	Mean+Std		p-value
	PD-ON	PD-OFF		PD-ON	PD-OFF		PD-ON	PD-OFF	
Jitter-abs ($10^{-5}$ sec)	7.22±6.34	8.31±9.23	0.484	5.87±5.87	4.76±4.07	0.013	6.21±5.17	11.42±13.48	0.000
Jitter-rel (%)	0.98±0.80	1.06±1.01	0.992	0.84±0.76	0.72±0.63	0.007	0.95±0.79	1.55±1.67	0.002
Jitter-rap (%)	0.56±0.49	0.61±0.62	0.992	0.48±0.48	0.41±0.38	0.040	0.52±0.48	0.92±1.07	0.000
Jitter-ppq5 (%)	0.59±0.51	0.61±0.58	0.806	0.49±0.41	0.40±0.36	0.003	0.58±0.47	0.87±0.85	0.007
Shimmer (dB)	1.08±0.47	1.04±0.45	0.599	0.95±0.45	0.86±0.48	0.017	0.95±0.48	1.09±0.57	0.006
Shimmer-rel (%)	12.07±5.42	11.72±5.27	0.764	10.48±5.14	9.69±5.57	0.051	10.55±5.60	12.35±6.59	0.000
Shimmer-apq3 (%)	6.29±2.98	6.09±2.79	0.775	5.38±2.89	5.18±3.23	0.260	5.29±3.02	6.58±3.82	0.000
Shimmer-apq5 (%)	7.93±3.93	7.69±4.02	0.870	6.92±3.61	6.22±3.71	0.017	6.82±4.23	7.83±4.56	0.015
Shimmer-apq11 (%)	8.62±3.51	8.74±3.75	0.560	7.91±3.79	6.94±3.70	0.005	7.81±4.18	8.38±4.33	0.241
Mean Pitch (Hz)	144.55±34.48	137.34±33.78	0.000	153.29±41.36	157.02±45.59	0.329	155.28±41.89	145.18±39.59	0.000
Median Pitch (Hz)	144.58±36.11	138.20±34.57	0.000	153.53±42.29	156.80±45.83	0.139	155.30±41.91	144.83±40.44	0.000
Std Pitch (Hz)	4.83±10.20	4.11±8.97	0.809	3.10±7.02	3.17±8.74	0.431	1.87±1.60	3.51±8.20	0.419
Min Pitch (Hz)	136.85±36.28	129.44±35.62	0.000	147.38±42.43	151.71±45.84	0.358	150.84±40.45	138.84±40.71	0.000
Max Pitch (Hz)	152.64±36.14	143.84±36.14	0.000	159.02±41.76	162.57±49.04	0.121	159.59±43.33	151.69±40.95	0.000
HNR (dB)	12.97±3.84	12.70±4.18	0.211	17.48±5.00	18.29±5.03	0.048	18.44±5.98	16.43±5.91	0.000
NHR	0.10±0.09	0.11±0.12	0.209	0.05±0.07	0.04±0.07	0.033	0.05±0.06	0.07±0.08	0.003

#### 2) Mann Whitney U-Test Between Co and PD-off

The Mann Whitney U-test results for group differences between CO and PD-*off* are demonstrated in Table 4. The table shows that there was a significant group difference between the majority of the voice features of all the phonemes except the shimmer of phoneme /a/.

**TABLE 4:** The Result of the Mann Whitney U Test Between CO and PD-OFF

Parameters	Phoneme /a/			Phoneme /o/			Phoneme /m/		
	Mean+Std		p-value	Mean+Std		p-value	Mean+Std		p-value
	CO	PD-OFF		CO	PD-OFF		CO	PD-OFF	
Jitter-abs ($10^{-5}$ sec)	4.16±3.22	8.31±9.23	0.000	3.80±4.19	4.76±4.07	0.000	5.13±5.39	11.42±13.48	0.000
Jitter-rel (%)	0.59±0.42	1.06±1.01	0.000	0.58±0.53	0.72±0.63	0.036	0.75±0.65	1.55±1.67	0.000
Jitter-rap (%)	0.33±0.26	0.61±0.62	0.000	0.33±0.33	0.41±0.38	0.050	0.43±0.40	0.92±1.07	0.000
Jitter-ppq5 (%)	0.34±0.26	0.61±0.58	0.000	0.33±0.27	0.40±0.36	0.042	0.45±0.35	0.87±0.85	0.000
Shimmer (dB)	1.03±0.54	1.04±0.45	0.643	0.75±0.49	0.86±0.48	0.017	0.91±0.54	1.09±0.57	0.003
Shimmer-rel (%)	11.69±6.22	11.72±5.27	0.659	8.41±5.50	9.69±5.57	0.013	10.28±6.15	12.35±6.59	0.003
Shimmer-apq3 (%)	6.11±3.65	6.09±2.79	0.534	4.26±3.18	5.18±3.23	0.002	5.20±3.31	6.58±3.82	0.001
Shimmer-apq5 (%)	7.47±4.16	7.69±4.02	0.567	5.39±3.66	6.22±3.71	0.022	6.73±4.06	7.83±4.56	0.056
Shimmer-apq11 (%)	8.35±3.88	8.74±3.75	0.350	6.41±4.09	6.94±3.70	0.045	7.78±5.00	8.38±4.33	0.060
Mean Pitch (Hz)	153.60±35.15	137.34±33.78	0.000	173.53±41.09	157.02±45.59	0.000	162.74±43.32	145.18±39.59	0.000
Median Pitch (Hz)	153.55±36.06	138.20±34.57	0.000	173.62±41.05	156.80±45.83	0.000	162.70±43.42	144.83±40.44	0.000
Std Pitch (Hz)	3.18±8.15	4.11±8.97	0.000	2.04±3.49	3.17±8.74	0.688	1.73±2.78	3.51±8.20	0.013
Min Pitch (Hz)	148.70±37.25	129.44±35.62	0.000	169.27±42.28	151.71±45.84	0.000	159.27±43.80	138.84±40.71	0.000
Max Pitch (Hz)	158.66±35.37	143.84±36.14	0.001	177.38±41.12	162.57±49.04	0.000	166.45±43.03	151.69±40.95	0.002
HNR (dB)	13.76±4.28	12.70±4.18	0.039	20.56±5.77	18.29±5.03	0.000	19.15±6.31	16.43±5.91	0.000
NHR	0.09±0.11	0.11±0.12	0.008	0.03±0.05	0.04±0.07	0.001	0.04±0.05	0.07±0.08	0.000

#### 3) Mann Whitney U-Test Between Co and PD-on

Table 5 gives the $p$-values for group differences between CO and PD-*on*. The results show that jitter, shimmer, and harmonics parameters of phoneme /o/ of the two groups were well separated. The jitter of phoneme /a/ and /m/ were effective to differentiate CO and PD-on.

**TABLE 5:** The Result of the Mann Whitney U Test Between CO and PD-ON

Parameters	Phoneme /a/			Phoneme /o/			Phoneme /m/		
	Mean+Std		p-value	Mean+Std		p-value	Mean+Std		p-value
	CO	PD-ON		CO	PD-ON		CO	PD-ON	
Jitter-abs ($10^{-5}$ sec)	4.16±3.22	7.22±6.34	0.000	3.80±4.19	5.87±5.87	0.000	5.13±5.39	6.21±5.17	0.001
Jitter-rel (%)	0.59±0.42	0.98±0.80	0.000	0.58±0.53	0.84±0.76	0.000	0.75±0.65	0.95±0.79	0.007
Jitter-rap (%)	0.33±0.26	0.56±0.49	0.000	0.33±0.33	0.48±0.48	0.000	0.43±0.40	0.52±0.48	0.018
Jitter-ppq5 (%)	0.34±0.26	0.59±0.51	0.000	0.33±0.27	0.49±0.41	0.000	0.45±0.35	0.58±0.47	0.009
Shimmer (dB)	1.03±0.54	1.08±0.47	0.324	0.75±0.49	0.95±0.45	0.000	0.91±0.54	0.95±0.48	0.403
Shimmer-rel (%)	11.69±6.22	12.07±5.42	0.398	8.41±5.50	10.48±5.14	0.000	10.28±6.15	10.55±5.60	0.643
Shimmer-apq3 (%)	6.11±3.65	6.29±2.98	0.303	4.26±3.18	5.38±2.89	0.000	5.20±3.31	5.29±3.02	0.713
Shimmer-apq5 (%)	7.47±4.16	7.93±3.93	0.205	5.39±3.66	6.92±3.61	0.000	6.73±4.06	6.82±4.23	0.806
Shimmer-apq11 (%)	8.35±3.88	8.62±3.51	0.370	6.41±4.09	7.91±3.79	0.000	7.78±5.00	7.81±4.18	0.490
Mean Pitch (Hz)	153.60±35.15	144.55±34.48	0.054	173.53±41.09	153.29±41.36	0.000	162.74±43.32	155.28±41.89	0.084
Median Pitch (Hz)	153.55±36.06	144.58±36.11	0.057	173.62±41.05	153.53±42.29	0.000	162.70±43.42	155.30±41.91	0.087
Std Pitch (Hz)	3.18±8.15	4.83±10.20	0.000	2.04±3.49	3.10±7.02	0.168	1.73±2.78	1.87±1.60	0.264
Min Pitch (Hz)	148.70±37.25	136.85±36.28	0.012	169.27±42.28	147.38±42.43	0.000	159.27±43.80	150.84±40.45	0.077
Max Pitch (Hz)	158.66±35.37	152.64±36.14	0.193	177.38±41.12	159.02±41.76	0.000	166.45±43.03	159.59±43.33	0.099
HNR (dB)	13.76±4.28	12.97±3.84	0.097	20.56±5.77	17.48±5.00	0.000	19.15±6.31	18.44±5.98	0.454
NHR	0.09±0.11	0.10±0.09	0.019	0.03±0.05	0.05±0.07	0.000	0.04±0.05	0.05±0.06	0.158

#### 4) Summary of the Statistical Analysis

Comparison of the results on the above statistical analysis, confirm that the majority of the parameters of the phoneme /o/ were significantly different between the healthy subjects (CO) and PD patients; both, PD-*on* and PD-*off*. Phoneme /m/ was effective to identify the change due to medication as well as differentiating between CO and PD-off. It is also seen that harmonics parameters of /o/ and /m/ were statistically different between PD-*off* and PD-*on*.

### B. Classification Analysis

The results of classification by SVM of the voice features of each of the three phonemes are shown in Table 6. It is seen that the best classification result between the three groups were obtained with the combination of the phonemes. The best AUC for the classification between PD-off and PD-*on* was 0.81. Classification between CO and PD-*off* shows that the best result was with the combination of the three phonemes, with AUC = 0.90. The best classification result between CO and PD-*on* was with the combination of phonemes /a/ and /m/ with AUC = 0.86.

**TABLE 6:** The SVM Classification Results With the Selected Parameters

Input to SVM (all parameters)	PD-on and PD-off				CO and PD-off				CO and PD-on			
	AUC	Acc (%)	FPR (%)	FNR (%)	AUC	Acc (%)	FPR (%)	FNR (%)	AUC	Acc (%)	FPR (%)	FNR (%)
/a/	0.68	61.6%	40.2%	35.7%	0.78	70.9%	30.0%	28.0%	0.74	67.9%	30.2%	32.9%
/o/	0.73	66.9%	36.2%	28.6%	0.79	69.1%	29.1%	31.4%	0.77	70.3%	37.1%	21.4%
/m/	0.73	70.8%	19.3%	38.5%	0.78	69.9%	32.0%	27.2%	0.85	77.5%	23.2%	21.8%
/a/ + /o/	0.74	65.5%	41.3%	27.9%	0.87	78.8%	25.2%	16.5%	0.83	73.7%	28.7%	23.9%
/a/ + /m/	0.79	71.3%	24.7%	32.1%	0.89	81.3%	17.7%	19.8%	0.86	78.1%	21.4%	22.0%
/o/ + /m/	0.75	67.8%	18.7%	44.3%	0.84	76.3%	19.1%	27.7%	0.83	74.3%	27.9%	22.4%
/a/ + /o/ + /m/	0.81	73.9%	19.1%	31.8%	0.90	79.7%	17.8%	23.4%	0.84	77.4%	22.5%	22.9%

## IV. Discussion

The statistical analysis in Table 3 shows that /m/ was the best performing individual phoneme in differentiating PD-*off* from PD-*on*. Time and amplitude perturbations (jitter and shimmer) decrease with medication, suggesting that levodopa improves voice quality. In differentiating control from PD-*off* recordings, in effect detecting parkinsonian dysarthria, all /a/, /o/ and /m/ achieved comparably good levels of statistical significance (Table 4) with the exception of shimmer of /a/. Table 5 shows a drop-off in significance of /m/ after medication, reflecting the shift of PD-*on* towards control values because of the better levodopa response for this phoneme. Table 6 presents the SVM classifications with a Gaussian kernel. SVM classifications reveal that the best results were obtained by combining the features for all three phonemes used in this study: /a/, /o/ and /m/. This can be seen for each of the comparisons, with the combined phonemes achieving AUCs between 0.81 and 0.90. SVM classification to separate control and PD-*on* with the phonemes /a/ and /m/ was slightly better than that of the three phonemes. Table 6 also shows that the ranking of the individual phonemes to separate control from PD values was consistent with those derived from Mann Whitney U testing in Tables IV and V, with the exception of the classification between control and PD-*on*. The explanation for these divergences could be that there are both linear and non-linear effects of the parkinsonian state on voice. The SVM used a Gaussian kernel and thus performed non-linear separation, in contrast to the linear statistical analysis of Tables 3–5.

The voice features that identify parkinsonian dysarthria are not exactly congruent with those that recognize the levodopa response in PD. This is relevant to the two different types of task for which computerized voice techniques might be used in research and clinical practice. One is the early detection of motoric evidence of PD in individuals at risk of developing the disorder. The other is the monitoring of treatment effects in established PD, either in the clinic or in drug trials. Nevertheless, for both of these purposes, we have demonstrated that combined analysis of a set of phonemes comprising /a/, /o/ and /m/ should give a satisfactory level of sensitivity.

Cusnie-Sparrow [31] found a significant change in percent and absolute shimmer when comparing control and PD patients. They reported that jitter and shimmer of the sustained phoneme /a/ demonstrated moderate correlations with perceived voice quality and showed sensitivity to medication. We suspect that some earlier studies that used only /a/ or /i/ to assess levodopa responsiveness may have performed better if other phonemes had been considered [34], [49], [50].

In our study, the features most significantly changed by medication were the time, amplitude, and harmonic perturbation of /m/. Of the three phonemes examined, /o/ probably requires the greatest aggregate control of muscles of articulation—precise positioning of the tongue at mid-height, with a rounded formation of the lips [51]. Some tongue control is required for /a/, with the lips open. For /m/, the lips are simply closed, tongue position is of little consequence, and air is passed through the nasal cavity. The relatively good response of /m/ to levodopa could imply that fine control of anterior articulatory muscles (of tongue, lips, and jaw) shows a degree of resistance to medication. There are many examples of uneven levodopa responsiveness in other aspects of parkinsonism. Gait freezing and postural instability can be refractory to drug treatment [52]. Levodopa improves speed and amplitude of finger tapping, but motor decrement shows little benefit [53]. Basic motor deficits of tremor and bradykinesia are often differentially affected by dopaminergic medication. Further research is needed to understand better the selective character of levodopa’s actions on voice production in PD. We recruited a group of patients at relatively early in their PD course (mean duration 5.3 years). Their MDS-UPDRS-III motor disability scores and degree of levodopa responsiveness were in keeping with this stage of the disease [54].

There are two novelties of this study. The first is that it has found that medication has a significant effect on the change of time and amplitude perturbation (jitter and shimmer) and harmonic to noise ratio of the phoneme /m/ which is confirmed also by SVM classification. Thus, /m/ can be used to differentiate between PD-*on* and PD-*off*. The second novelty is that this study confirms that the three groups can be differentiated best when the three phonemes, /a/, /o/ and /m/, are used.

The limitation of this study is that because of the relatively small number of participants, it was not possible to differentiate between genders. Further, the differences such as accents, demographics, and language skills have not been investigated. Another shortcoming of this study is that each participant was only studied once and hence the repeatability has not been checked. Recruitment of controls was subject to some conditions from Research Ethics approval to advertising, and the mean control group age is about 5 years younger than the PD patients.

## V. Conclusion

This study has investigated the effect of levodopa medication on the voice of PD patients based on utterance of three phonemes, /a/, /o/ and /m/. It has found that medication has a significant effect on the change of time and amplitude perturbation (jitter, shimmer and harmonic to noise ratio) of the phoneme /m/. But the highest accuracy in differentiating PD-*on* and PD-*off*, using SVM, was when all three phonemes were used. While /o/ showed the greatest differences between PD and controls, the best classifications when using SVM were again obtained from combined analysis of all three phonemes. Whether attempting to separate parkinsonian dysarthria from control voice, or to detect the levodopa effect on voice in PD, this study shows that computerized analysis of multiple phonemes should be employed. Our findings are potentially relevant in research to identify early parkinsonian dysarthria, and for tele-monitoring of the levodopa response in patients with established PD.
